# A Model of Two-Way Selection System for Human Behavior

**DOI:** 10.1371/journal.pone.0081424

**Published:** 2014-01-13

**Authors:** Bin Zhou, Shujia Qin, Xiao-Pu Han, Zhe He, Jia-Rong Xie, Bing-Hong Wang

**Affiliations:** 1 Department of Modern Physics and Nonlinear Science Center, University of Science and Technology of China, Hefei, China; 2 Suzhou Institute of Technology, Jiangsu University of Science and Technology, Suzhou, China; 3 State Key Lab of Robotics, Shenyang Institute of Automation, Chinese Academy of Sciences, Shenyang, China; 4 Institute of Information Economy and Alibaba Business College, Hangzhou Normal University, Hangzhou, China; 5 College of Physics and Electronic Information Engineering, Wenzhou University, Wenzhou, China; University of Maribor, Slovenia

## Abstract

Two-way selection is a common phenomenon in nature and society. It appears in the processes like choosing a mate between men and women, making contracts between job hunters and recruiters, and trading between buyers and sellers. In this paper, we propose a model of two-way selection system, and present its analytical solution for the expectation of successful matching total and the regular pattern that the matching rate trends toward an inverse proportion to either the ratio between the two sides or the ratio of the state total to the smaller group's people number. The proposed model is verified by empirical data of the matchmaking fairs. Results indicate that the model well predicts this typical real-world two-way selection behavior to the bounded error extent, thus it is helpful for understanding the dynamics mechanism of the real-world two-way selection system.

## Introduction

Human-initiated systems always run in a complex way. In the past ten years, related work mainly focused on the temporal and spatial distribution characteristics of human activity patterns. Because of the complexity of human behavior, many underlying mechanisms have not been discovered yet. The two-way selection scenario among humans is one of the complicated but common phenomena in daily life. It happens in the processes like choosing a mate between men and women, making contracts between job hunters and recruiters, and trading between buyers and sellers. In a sense, two-way selections can be regarded as the base of building many social relationships. Generally, the participants in a two-way selection process are first classified into two groups by their natural status. Then they observe, study the factors of the people on the other side, and finally make their choices. For instance, in the case of marriages, one's appearance, personality, wealth, and sense of humor, are prevalently taken into consideration. Besides the individual characters, impersonal factors also exert an influence, e.g. the member totals on each side and their ratio. How many characters will be inspected and chosen deeply affects the result of a selection process. However, usually it is difficult to compare and to distinguish these characteristics quantitatively even qualitatively through traditional methods, such as psychological tests and social surveys.

The well-known marriage game in statistical physics has been researched in these papers [1]–[6], whose main novel concept is the stability of marriages. This view point aims to find a stable matching between the two sets of men and women. Such a model results in the destiny that every one in the sets gets married and the final marriage relationships are “stable”. However, the internal mechanism of a two-way selection system can be modeled in another way: not all of the participants have to get married in one trial of the processes, i.e. some of them would be successful in matching but the others not. This mechanism would render assistance to some social problems, such as the prediction of the total of friendships or other gregarious relations [7]–[15]. In this paper, we present a model for two-way selections to investigate the factors influencing the matching rate. The data of matchmaking fairs are analyzed to support our model. Based on this model, the method of estimating the number of factors impacting people's decisions is also proposed.

## The Model and Analytical Results

Our model of the two-way selection is stated as follows:

- The system has two sets of agents, *A* and *B*, respectively amounting to 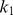 and 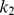.
- The *i*th agent in set *A* (or set *B*) has its own character denoted by 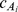 (or 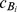). Correspondingly, the character the *i*th agent attempts to select is denoted by 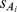 (or 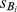).
- The agents' characters are denoted by integers without loss of generality. Assume the characters has *n* types, i.e. 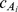, 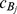, 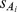, 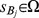, 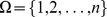, 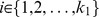, 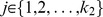. In one trial of the model, 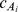, 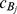, 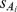, 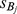 pick an element in *S* following the uniform distribution.
- The condition of successful matching of two agents 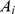 and 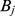 is 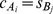 and 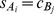. That is, when agent 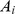
's character meets agent 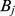
's requirement and *vice versa*, agent 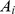 and agent 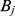 have a successful matching.

For given 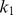, 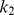 and *n*, the expectation *E* of the total number of matching pairs in the model is
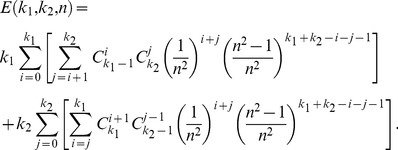
(1)According to
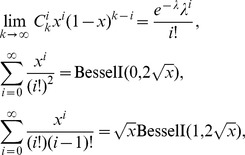
where 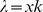, 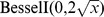 and 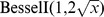 are the modified Bessel functions of the first kind, the expectation in (1) can approximate to
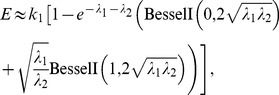
(2)where 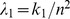, 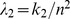.

Due to the symmetry of 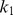 and 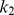 in (1), without loss of generality, we just study the 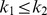 case under three conditions: 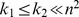, 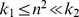, and 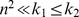. When 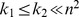, resulting in 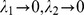, calculating the zeroth power term and the first power term of (2) obtains
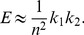
(3)When 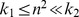 or 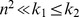, according to
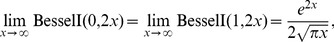
(4)
Equation (2) can be simplified as
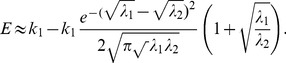
(5)Because in this case 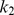 is very large, Equation (5) can be further simplified as
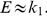
(6)


Define
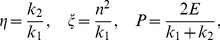
(7)where *η* denotes the ratio of 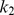 to 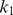; 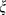 denotes the ratio of 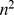 to 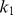; *P* denotes the estimated ratio of successful matching pairs to the average number of two type agents. Then Equation (2) can be transformed into
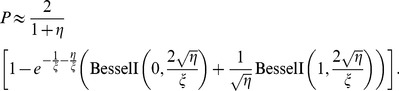
(8)
Equation (3) can be written as:
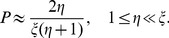
(9)
Equation (6) can be written as:
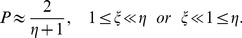
(10)


**Figure 1:**
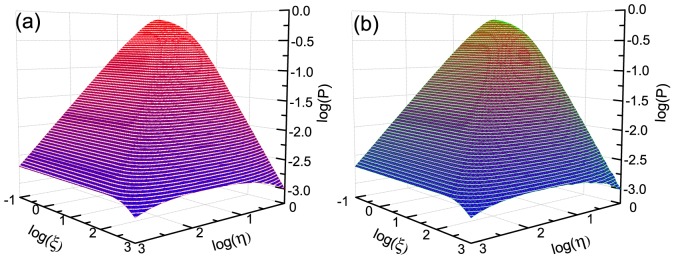
The comparison between the analytical predictions and the simulation results. In the two sub-figures, the parameter 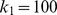; *η* is assigned to values from 1 to 1000; 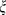 is assigned to values from 0.1 to 1000. (a) shows analytical predictions of (1); (b) shows the simulation results.

**Figure 2:**
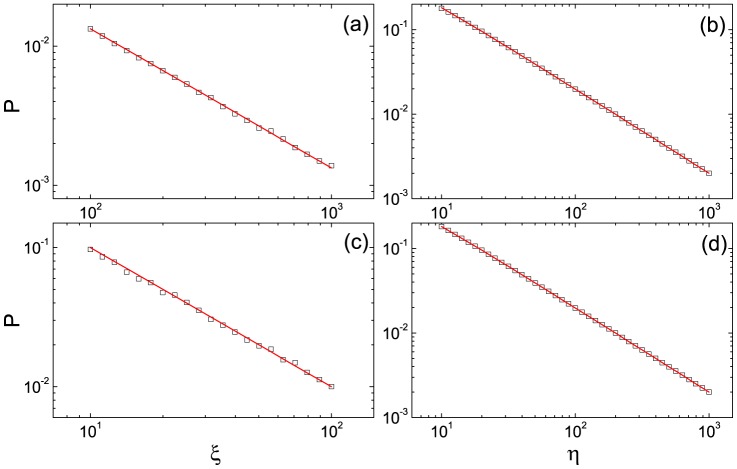
The comparison between analytical predictions and the simulation results in the log-log plots. In the four sub-figures, the parameter 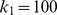; the squares are the simulation data; the solid lines are analytical predictions. In (a), 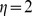; 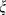 is assigned to values from 100 to 1000; the solid line is obtained from (9). In (b), 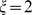; 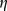 is assigned to values from 10 to 1000; the solid line is obtained from (10). In (c), 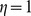; 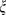 is assigned to values from 10 to 100; the solid line is obtained from (11). In (d), 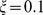; *η* is assigned to values from 10 to 1000; the solid line is obtained from (10).

Figure 1 shows the comparison between the analytical predictions of (1) and the simulation results. Figure 2(a) shows the comparison between the analytical predictions of (9) and the simulation results under the condition 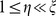 and displays a power-law relation with the exponent −1 between *P* and 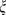. Figure 2(b) shows the comparison between the analytical predictions of (10) and the simulation results under the condition 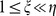 and displays a power-law relation with the exponent −1 between *P* and *η*; Figure 2(d) shows the comparison between the analytical predictions of (10) and the simulation results under the condition 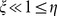 and displays the same power-law relation to the result in Figure 2(b). The above analytical predictions and simulation results are consistent with each other. That is to say all analytical results are reliable.

Consider a special case 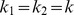, resulting in 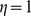. On the one hand, Equation (9) can be simplified as
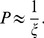
(11)The relation between *P* and 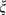 approximates a power law with the exponent −1, and this case is shown in Figure 2(c). Equation (10) can be simplified as 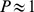, suggesting that almost all of the agents can match successfully under the condition 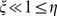. On the other hand, because the condition 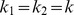 results in 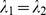, from (5) we can obtain
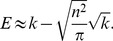
(12)The second term of (12) is the number of the agents that can not successfully match in type A or type B. The larger *k* is, the smaller 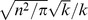 is. In reality, this is a result of fluctuation. The total combinations of the “own” state and the “expecting” state for an agent have 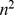 possibilities in the model. In theory, the expected times of each state appearance is 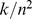. However, due to the fluctuations, almost all frequencies of every state appearance deviate around 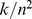. As a result, some agents can not successfully match. The number of times that each state may appear obeys the binomial distribution. The fluctuation is closely related to the standard deviation, according to the binomial theorem and standard deviation formula, we can obtain that the standard deviation equals 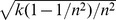, which is directly proportional to 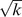. Thus, the number of the agents that can not successfully match is also proportional to 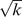. It explains the relationship between the second item of (12) and 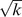. From (12), we know that the proportionality coefficient is 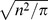.

## The Verification Between the Model and Experimental Data

As the mate choosing between men and women is a typical real-world two-way selection system, eighty-two reported records of matchmaking fairs are analyzed to verify our model. Due to the uncertainty of approximation in these reports, we classify the data into three categories with specified possible ranges according to their descriptions: i) “nearly *x*” (possible range 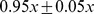); ii) “about *x*” (possible range 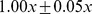); iii) “over *x*” (possible range 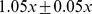). The full list of the data records is shown in Table 1. All data of matchmaking fairs are collected from the websites shown in Table 2.

**Table 1:** The data of matchmaking fairs.

Website no.	Original descriptions		Total participants *K*	Matched pairs *E*	Matching ratio *P*
	joined	matched			
01	13	3	13	3	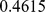
02	18	6	18	6	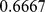
03	20	1	20	1	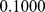
04	21	6	21	6	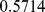
05	25	5	25	5	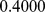
06	26	3	26	3	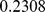
07	26	4	26	4	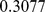
08	30	2	30	2	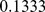
09	32	4	32	4	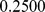
10	36	6	36	6	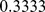
11	36	6	36	6	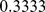
12	38	6	38	6	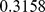
13	40	8	40	8	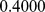
14	>40	3	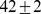	3	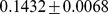
15	>50	3	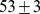	3	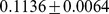
16	≈60	5	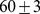	5	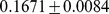
17	>60	4	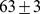	4	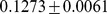
18	>60	5	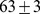	5	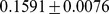
19	>60	≈10	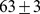	10±1	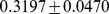
20	72	11	72	11	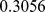
21	80	5	80	5	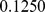
22	80	10	80	10	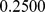
23	80	13	80	13	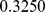
24	80	18	80	18	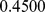
25	∼100	5	95±5	5	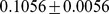
26	99	8	99	8	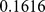
27	100	5	100	5	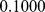
28	≈100	7	100±5	7	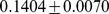
29	>100	16	110±10	16	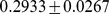
30	150	7	150	7	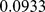
31	∼200	8	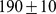	8	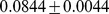
32	∼200	22	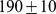	22	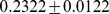
33	≈200	4	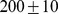	4	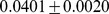
34	206	10	206	10	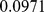
35	>200	7	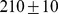	7	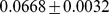
36	>200	8	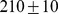	8	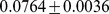
37	>200	38	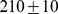	38	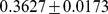
38	216	19	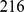	19	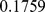
39	>240	22	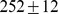	22	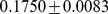
40	≈258	>10	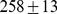	11±1	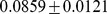
41	∼300	4	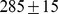	4	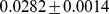
42	≈300	8	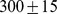	8	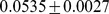
43	>300	>10	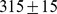	11±1	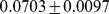
44	>300	32	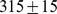	32	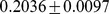
45	400	∼20	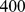	19±1	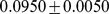
46	>500	3	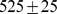	3	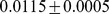
47	>500	8	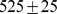	8	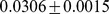
48	>500	>10	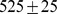	11±1	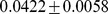
49	∼600	∼40	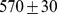	38±2	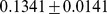
50	>600	>78	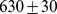	78	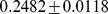
51	∼800	58	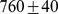	58	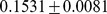
52	>800	>20	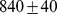	21±1	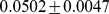
53	∼1000	≈20	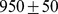	20±1	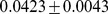
54	∼1000	58	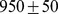	58	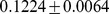
55	∼1000	64	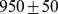	64	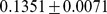
56	≈1000	12	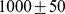	12	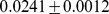
57	≈1000	15	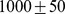	15	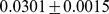
58	≈1000	∼100	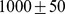	95±5	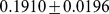
59	>1000	3	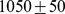	3	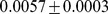
60	>1000	4	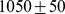	4	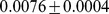
61	>1500	48	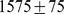	48	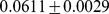
62	>1500	>100	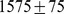	105±5	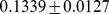
63	>1600	31	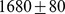	31	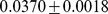
64	>2000	∼100	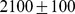	95±5	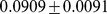
65	>2000	>113	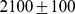	119±6	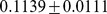
66	∼3000	∼100	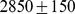	95±5	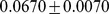
67	≈3000	186	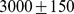	186	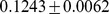
68	>3000	>200	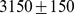	210±10	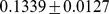
69	>4000	>500	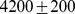	525±25	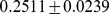
70	∼5000	108	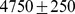	108	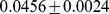
71	>5000	218	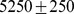	218	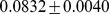
72	>5000	231	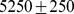	231	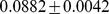
73	>5000	237	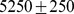	237	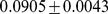
74	>6000	>270	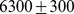	284±14	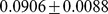
75	∼10000	28	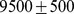	28	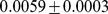
76	∼10000	≈100	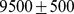	100±5	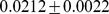
77	∼10000	∼2000	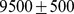	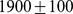	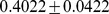
78	>10000	≈400	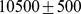	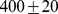	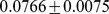
79	>10000	>1000	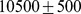	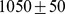	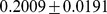
80	≈16000	>700	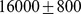	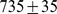	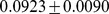
81	>16000	>600	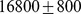	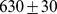	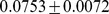
82	>50000	>3000	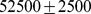	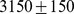	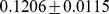

Note: ≈ denotes “about”, ∼ denotes “nearly”, and > denotes “above”.

**Table 2:** Data sources of matchmaking fairs.

Website no.	Website name of matchmaking fairs
01	http://www.cdb.org.cn/newsview.php?id=6359
02	http://sd.people.com.cn/n/2012/0827/c183718-17407663.html
03	http://news.carnoc.com/list/183/183765.html
04	http://bbs.tiexue.net/post2_5756757_1.html
05	http://www.shxb.net/html/20110516/20110516_278862.shtml
06	http://news.qq.com/a/20100511/000472.htm
07	http://bbs.ganxianw.com/thread-46316-1-1.html
08	http://www.wzrb.com.cn/article321273show.html
09	http://www.wccdaily.com.cn/epaper/hxdsb/html/2012-05/14/content_448239.htm
10	http://wbnews.sxrb.com/news/ty/1372966.html
11	http://www.nbmz.gov.cn/view.aspx?id=16595&AspxAutoDetectCookieSupport=1
12	http://nb.people.com.cn/GB/200892/16491824.html
13	http://cq.cqwb.com.cn/NewsFiles/201203/25/921397.shtml
14	http://www.sc.chinanews.com.cn/my/data/html/201212/32619.html
15	http://www.16466.com/info_detail.htm?id=36526
16	http://www.ncnews.com.cn/ncxw/shxw/t20121112_943114.htm
17	http://news.wzsee.com/2012/0502/130061.html
18	http://news.hexun.com/2012-08-27/145162214.html
19	http://www.douban.com/group/topic/28145139/
20	http://zhuanti.10yan.com/zt/other/sdcms/html/xqj2012/xiangqindongtai/1377.html
21	http://www.wlmqwb.com/3229/syzt/hdzt/seven/201007/t20100719_1287834.shtml
22	http://www.dpcm.cn/html/news/shehui/20121211/8a485b96f289e038.htm
23	http://www.zhaogejia.com/News/Show/166
24	http://cq.cqnews.net/shxw/shwx/200909/t20090928_3635782.htm
25	http://a.jiaodong.net/jiaoyou/detail/?/20120717134715.htm
26	http://www.dllake.com/testurl/news/news.asp?id=1874
27	http://fj.qq.com/a/20120413/000073.htm?pgv_ref=aio2012&ptlang=2052
28	http://epaper.lnd.com.cn/html/bdcb/20110118/bdcb635730.html
29	http://news.zh853.com/NewsShow-22166.html
30	http://news.163.com/11/1123/08/7JHJ4PP100014AED.html
31	http://news.xinmin.cn/rollnews/2011/05/03/10539635.html
32	http://www.0523qq.com/forum.php?mod=viewthread&tid=2779
33	http://news.ycw.gov.cn/html/2012-04/28/content_15150376.htm
34	http://epaper.lnd.com.cn/html/bdcb/20110118/bdcb635730.html
35	http://www.cqwb.com.cn/NewsFiles/201005/30/20102930062910354716.shtml
36	http://bddsb.bandao.cn/data/20120827/html/53/content_2.html
37	http://www.zhaogejia.com/News/Show/150
38	http://3g.3xgd.com/news/play.asp?NewsID=80975
39	http://wed.cnhan.com/hjb/2012-12-03/3900.html
40	http://xt.fangyuan365.com/article/List.asp?ID=8708
41	http://cq.cqnews.net/shxw/2012-11/12/content_21432170.htm
42	http://xt.fangyuan365.com/article/List.asp?ID=11694
43	http://roll.sohu.com/20120625/n346389451.shtml
44	http://www.ijxjj.com/article/article_12773.html
45	http://news.xinmin.cn/shehui/2013/02/16/18638248_2.html
46	http://www.sz120.com/xwdt/ynxw/22205/
47	http://www.sc.xinhuanet.com/content/2012-02/06/content_24649397.htm
48	http://dqnews.zjol.com.cn/dqnews/system/2010/08/17/012525402.shtml
49	http://cheshang.16888.com/newsinfo/2011/1115/141264.html
50	http://bbs.heze.cc/thread-842865-1-1.html
51	http://www.8hy.org/hyjy/hy6240/1
52	http://www.cdrb.com.cn/html/2012-04/03/content_1546492.htm
53	http://heilongjiang.dbw.cn/system/2013/02/16/054584150.shtml
54	http://www.e0734.com/2012/0502/90707.html
55	http://sz.tznews.cn/tzwb/html/2012-07/09/content_71285.htm
56	http://www.xtrb.cn/epaper/ncwb/html/2011-08/09/content_275667.htm
57	http://www.hukou365.com/cwbbs/forum/showtopic_tree.jsp?rootid=194730
58	http://news.163.com/10/0329/03/62TPSMMO000146BB.html
59	http://www.chinajilin.com.cn/content/2009-02/15/content_1495554.htm
60	http://sy.house.sina.com.cn/news/2011-12-27/114483993.shtml
61	http://news.dayoo.com/guangzhou/201205/02/73437_23554932.htm
62	http://cq.qq.com/a/20090824/000190.htm
63	http://news.qq.com/a/20111228/000342.htm
64	http://www.efu.com.cn/data/2011/2011-08-09/389729.shtml
65	http://ent.163.com/12/1203/13/8HQ885MN00032DGD.html
66	http://www.subaonet.com/html/society/2010426/3C95FFIB98JI5FC.html
67	http://wb.sznews.com/html/2011-11/07/content_1812730.htm
68	http://heilongjiang.dbw.cn/system/2012/04/23/053819817.shtml
69	http://news.cnnb.com.cn/system/2011/10/31/007128083.shtml
70	http://www.people.com.cn/GB/paper447/17168/1505082.html
71	http://news.timedg.com/2012-04/16/content_9577975.htm
72	http://www.gddgart.com/artcenter/html3asp/town3ship/dq2012714_2357.asp
73	http://epaper.oeeee.com/I/html/2012-11/12/content_1751538.htm
74	http://news.hsw.cn/system/2010/06/28/050547974.shtml
75	http://fj.sina.com.cn/news/s/2012-08-24/07186785.html
76	http://net.chinabyte.com/164/12210164.shtml
77	http://epaper.hljnews.cn/shb/html/2008-05/26/content_199685.htm
78	http://www.estour.gov.cn/news/lvyouxinwen/2011/815/1181583840H7H40DE3ADE501J93BG9.shtml
79	http://www.048100.com.cn/news/bdxw/2009-04-20/617.html
80	http://www.estour.gov.cn/news/lvyouxinwen/2011/815/1181583840H7H40DE3ADE501J93BG9.shtml
81	http://www.estour.gov.cn/news/lvyouxinwen/2011/815/1181583840H7H40DE3ADE501J93BG9.shtml
82	http://zjnews.zjol.com.cn/05zjnews/system/2009/03/30/015385573.shtml

In our model, *n* is an internal parameter needed to be measured. Because a news report (descried as an experiment below) generally includes only the total of participants and the number of successful matching pairs, the male–female or female–male ratio *η* defined in (7) should be estimated first. Under the assumption 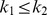, the lower bound of *η* is 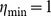, and once the total of participants 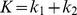 and the number of matching couples *E* is determined, the upper bound of *η* in that experiment is known: 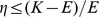. Let *N* be the number of experiments, 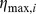 be the upper bound of *η* in the *i*th experiment, and 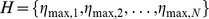 be the set of all upper bounds. By processing 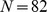 experiments in Table 1, we obtain 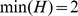 and 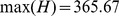. Consider the least square criterion for fitting the model and the experimental data
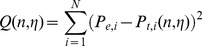
(13)where 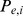 denotes the experimental data in the *i*th experiment and 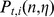 denotes the corresponding theoretical value calculated by (8), and the reality that in a matchmaking fair the numbers of males and females would not differ over some extent. We narrow the range of *η* to [1], [2] and solve this optimization problem
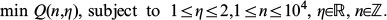
(14)Finally we obtain the estimation 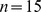.

**Figure 3:**
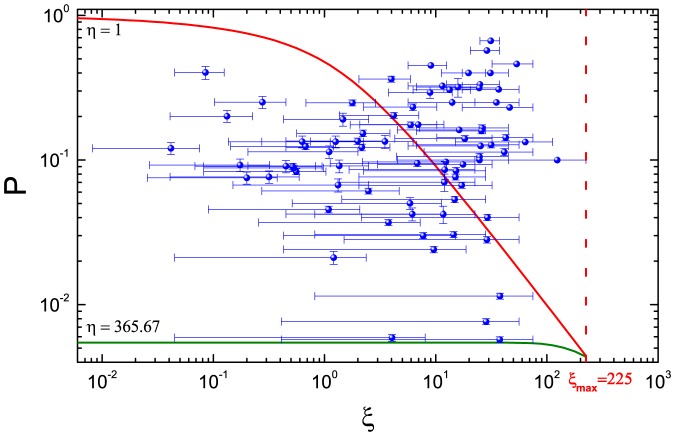
The relationship between the experimental data and analytical predictions in the log-log plots. The red curve and the olive curve are obtained from (8), and the parameters of red curve are 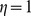, 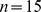; The parameters of olive curve are 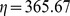, 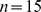. The round dots represent the empirical data in Table 1. The 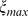 represents the maximum value 225 of 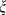.

Figure 3 shows the relationship between the experimental data and the analytical predictions of our model. The red curve and olive curve are obtained from (8). The parameters of red curve are 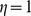, 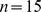; the parameters of olive curve are 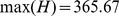, 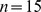. According to (7), when 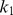 is equal to the minimum 1, 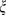 takes the maximum value 225. The error bars of ordinate *P* of round dots represent the ranges of empirical data *P* in Table 1. Because 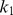 is unknown and 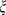 is undetermined, the bound for 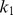 in the *i*th experiment is 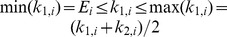, and the bound for corresponding 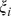 is 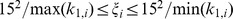. Therefore, the ranges of abscissa 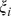 of round dots are relatively wide and the middle points lie in 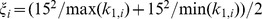.

Figure 3 also shows when 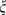 is relatively small and corresponding 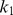 is big, all empirical data are enclosed between two curves; when 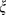 is relatively big and corresponding 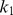 is small, some empirical data are enclosed between the two curves, but other empirical data lie above the red curve and the trend of the empirical data is opposite to the analytical predictions. The possible reasons are: on the one hand, organizers of some matchmaking fairs select only a few participants meeting their requirements from a large number of applicants, so a participant is easier to find the right man or woman; on the other hand, when the number of participants is small in a matchmaking fair, they understand the difficulty of finding an ideal object so compromise to a goodish choice. The two reasons above cause that the fewer the participants are, the higher the matching probability *P* is. Based on these effects, the deviation of experimental data from the model is acceptable.

## Conclusion

We propose a model of the two-way selection system and provide its analytical solution. Under several conditions, the compact approximations are derived analytically and verified by the simulation results. In the model, the parameter *n* that denotes the number of characters directly determines the probability of the successful match – due to its importance, we propose a rough method to estimate its value by fitting the empirical data collected via the Internet and the result is 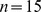. Under some artificial assumptions, most of the experimental data fall into the range predicted by our model, so this model is helpful for understanding the dynamics mechanism of the real-world two-way selection systems, and provides a starting point for researching the nature of real-world two-way selection systems. We believe our model could enlighten readers in this rapidly developing field.
